# Mechanical Properties, Biodegradation, and Biocompatibility of Ultrafine Grained Magnesium Alloy WE43

**DOI:** 10.3390/ma12213627

**Published:** 2019-11-04

**Authors:** Sergey Dobatkin, Natalia Martynenko, Natalia Anisimova, Mikhail Kiselevskiy, Dmitriy Prosvirnin, Vladimir Terentiev, Nikita Yurchenko, Gennady Salishchev, Yuri Estrin

**Affiliations:** 1 A.A. Baikov Institute of Metallurgy and Materials Science of the RAS, Moscow 119334, Russia; dobatkin.sergey@gmail.com (S.D.); imetran@yandex.ru (D.P.); fatig@mail.ru (V.T.); 2 National University of Science and Technology “MISIS”, Moscow 119049, Russia; n_anisimova@list.ru (N.A.); kisele@inbox.ru (M.K.); 3 N.N. Blokhin National Medical Research Center of Oncology of the Ministry of Health of the Russian Federation, Moscow 115478, Russia; 4 Belgorod National Research University, Belgorod 308015, Russia; yurchenko_nikita@bsu.edu.ru (N.Y.); salishchev@bsu.edu.ru (G.S.); 5 Department of Materials Science and Engineering, Monash University, Melbourne 3800, Australia; yuri.estrin@monash.edu; 6 Department of Mechanical Engineering, The University of Western Australia, Nedlands 6907, Australia

**Keywords:** biomedical materials, magnesium alloys, multiaxial deformation, ultrafine grain structure, mechanical properties, fatigue strength, biodegradation, biocompatibility

## Abstract

In this work, the effect of an ultrafine-grained (UFG) structure obtained by multiaxial deformation (MAD) on the mechanical properties, fatigue strength, biodegradation, and biocompatibility in vivo of the magnesium alloy WE43 was studied. The grain refinement down to 0.93 ± 0.29 µm and the formation of Mg_41_Nd_5_ phase particles with an average size of 0.34 ± 0.21 µm were shown to raise the ultimate tensile strength to 300 MPa. Besides, MAD improved the ductility of the alloy, boosting the total elongation from 9% to 17.2%. An additional positive effect of MAD was an increase in the fatigue strength of the alloy from 90 to 165 MPa. The formation of the UFG structure also reduced the biodegradation rate of the alloy under both in vitro and in vivo conditions. The relative mass loss after six weeks of experiment was 83% and 19% in vitro and 46% and 7% in vivo for the initial and the deformed alloy, respectively. Accumulation of hydrogen and the formation of necrotic masses were observed after implantation of alloy specimens in both conditions. Despite these detrimental phenomena, the desired replacement of the implant and the surrounding cavity with new connective tissue was observed in the areas of implantation.

## 1. Introduction

The study of magnesium alloys aiming at their medical applications has become an integral part of modern materials science. Magnesium is an excellent medical material due to its high specific strength and good biocompatibility [1,2,3,4,5]. The flip side of Mg alloys is their excessively high degradation rate in bodily fluids. Besides, the degradation process is accompanied with the evolution of hydrogen gas. The released hydrogen is accumulated in the tissues around the implant, which slows down the healing process and may lead to complications [6]. Therefore, one of the main tasks in the development of magnesium-based implants is the reduction of their degradation rate, which leads, among other things, to a decrease in the rate of gas formation. Alloying of magnesium with various metals is used to allay this disadvantage. A crucial consideration is that, while having a positive effect on the corrosion resistance, the selected alloying elements should not have a pronounced negative effect on the vital activity of cells. Therefore, the number of suitable metal elements is small [7]. Currently, metals essential for human body as trace elements (e.g., Ca and Zn) are used as the main alloying elements. However, some rare earth metals (Y, Nd, and Dy) are also being considered since they have a positive effect on the corrosion resistance of magnesium and, according to the available data, do not show significant toxicity to living cells. To that end, long-known commercial alloys are adapted to the targeted use in implants and new compositions are developed. Among these alloys, the magnesium alloy WE43 of the Mg-Y-Nd-Zr system is one of the most popular ones [8]. The presence of rare-earth metals significantly increases both the corrosion resistance of the alloy and its mechanical strength. Zirconium acts as a modifier for grain refinement and in addition increases the strength of the alloy. However, solid solution strengthening of alloy WE43 cannot provide the level of mechanical properties required in orthopedic implants. There are two ways to enhance the strength characteristics of the alloy: through precipitation of intermetallic particles during artificial aging or through grain refinement by plastic deformation. The first method leads to an increase in the strength of WE43, but, at the same time, significantly reduces ductility [9], which limits its further application. Therefore strain hardening by plastic deformation remains a viable option. Deformation methods that allow refining the microstructure down to an ultrafine-grained (UFG) state, such as severe plastic deformation (SPD) techniques [10] come to the fore. It was shown that it is possible to obtain an ultrafine-grained structure in magnesium alloys by SPD methods, including high-pressure torsion [9] and equal-channel angular pressing [11], as well as by more traditional metal forming techniques, such as multiaxial deformation [12], rotary swaging [13], or radial shear rolling [14]. In some cases it was shown that by using SPD techniques strengthening of magnesium alloys can be achieved without compromising their corrosion resistance and in some cases even with a side benefit of enhancing it [15,16,17].

One of the most interesting methods for obtaining the UFG state in metals and alloys is multiaxial deformation (MAD) or, as it is often called, multistep isothermal forging. This procedure includes a set of operations of upsetting, canting, and drawing, as a result of which an effective grain refinement occurs. This procedure has been widely used, for example, to improve the properties of aluminum [18,19] and titanium [20] alloys. In addition, the effectiveness of using this processing method for grain refinement in magnesium alloys down to the UFG state was demonstrated in [12,21,22,23,24,25,26,27,28,29,30]. Thus, Li et al. [26] showed that the application of MAD to alloy Mg-2%Zn-2%Gd allows attaining a very fine microstructure with the average grain size of ~500 nm. This can be seen as indication that it should be possible to produce an UFG structure in magnesium alloys alloyed with rare-earth metals by using MAD. In this case, the structure caused by MAD is rather homogeneous, which positively affects the stability of the final properties. It is also worth noting that the effect of MAD on the structure and properties of alloy WE43 was already considered in [12,21,29,30]. However, according to these articles, the authors failed to achieve the formation of a UFG structure in the alloy investigated. The grain size attained through MAD processing in these cases was 4.8 µm [12] and 6 µm [30]. This relatively low degree of grain refinement can be associated with the high processing temperature, which prevented the accumulation of a sufficiently high dislocation density. Still, a significant strengthening effect was observed after MAD. We note that in the cited works the alloy was not studied in terms of its potential use as a medical implant material.

In addition to a positive effect on the strength characteristics of magnesium alloys, as noted above, SPD often does not impair their corrosion resistance [15,16,17]. Thus, it was shown for a Mg-1Ca alloy that the formation of a UFG structure after high pressure torsion (HPT) leads to a significant increase in the corrosion resistance of the alloy in Ringer’s solution [31]. The authors argued that the mechanism of increased corrosion resistance is based on a balance between the processes of anodic dissolution of the Ca-containing secondary phase and deposition of hydroxide compounds on the specimen surface, which inhibits corrosion. A decrease in the particle size of the secondary Mg_2_Ca phase and a more uniform distribution of particles caused by HPT can also lead to a decrease of the biodegradation rate. Hosaka et al. [32] reported that the temperature of equal-channel angular pressing (ECAP) had a strong effect on the degradation rate of magnesium alloy AZ31. They showed that degradation products formed on the specimen surface provide an effective barrier to corrosion. It should also be noted that in addition to diminishing the overall corrosion rate ECAP-induced grain refinement of AZ31 reduces the propensity for pitting corrosion [33]. It can be assumed that by finding an appropriate SPD processing temperature microstructure refinement, uniform precipitation of fine dispersed particles and a reduced density of the crystal lattice defects can be achieved. A combination of these factors may play out to enhance the corrosion resistance of magnesium alloys. In addition, a decrease of the biodegradation rate will bring about an improvement of the biocompatibility of the material due to a slower rise of the pH value of the medium.

In earlier work, we tested the biocompatibility of magnesium alloy WE43 in homogenized condition in vitro and in vivo and assessed its biodegradation rate in a standard simulated body fluid represented by fetal bovine serum [34]. Based on the level of hemolysis and cytotoxicity, the alloy was categorized as a biocompatible one. Other researchers came to a similar conclusion [35]. These data motivate further investigation of the biocompatibility of the SPD-strengthened alloy WE43.

The aim of the present work was to study the effect of multiaxial deformation on the behavior of magnesium alloy WE43 from the viewpoint of its suitability for medical implant applications. For this purpose, in addition to the effect of MAD on the microstructure and tensile properties of the alloy, its fatigue strength, biodegradation, and biocompatibility in vivo was conducted. The resulting property profile, which is of interest for application of the alloy in medical implants, is reported below.

## 2. Materials and Methods 

**Figure 1:**
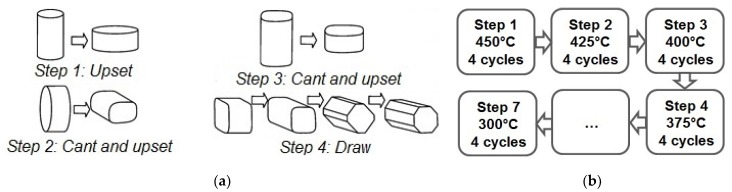
Schematics of multiaxial deformation process (**a**) and the processing schedule (**b**).

We used a commercial magnesium alloy WE43 containing, according to chemical analysis, 3.56% Y, 2.20% Nd, and 0.47% Zr (wt%) as a material for study. The cast alloy was homogenized at 525 °C for 8 h, then extruded at 430 °C with an extrusion ratio of 6.6 to give the work piece the desired shape and annealed again at 525 °C for 8 h. This was followed by air cooling. The cooling rate was sufficient to obtain a supersaturated solid solution in this alloy. Henceforth, the state of the alloy after this treatment will be referred to as the initial state. Multiaxial deformation was carried out on cylindrical specimens with a diameter of 25 mm and a length of 40 mm in multiple (up to 28) passes. This corresponds to a cumulative equivalent true strain of 17.5. This was accompanied with a step-wise decrease in the processing temperature from 450 °C to 300 °C (Figure 1). The deformation was carried out on an Instron 300LX (Instron, High Wycombe, UK) universal hydraulic static testing machine with a deformation rate of 2 mm/min.

The structure of the alloy in the initial state was studied by optical microscopy using an Axio Observer. D1m optical microscope (Carl Zeiss, Jena, Germany) equipped with Tixomet software (Version Pro). The structure after deformation processing was studied by transmission electron microscopy (TEM) using a JEM-1400 electron microscope (Jeol, Tokyo, Japan) operating at a voltage of 120 kV. Foils for TEM experiments were mechanically thinned and subjected to ion-bombardment on a GATAN 600 unit (GATAN Technologies, Pleasanton, USA). The size of the structural entities (such as grain or dislocation cell size) was estimated using Image Expert Professional 3 software (Version 3, Moscow, Russia).

The mechanical properties of the alloy were evaluated using uniaxial tensile tests carried out at room temperature in an Instron 3382 testing machine (Instron, High Wycombe, UK) with an extension rate of 1 mm/min. Cylindrical specimens with the diameter of 3 mm, the total length of 33 mm and the gage length of 15 mm were used. For fatigue tests, specimens with a cross-section of 1 × 1 mm and a length of 4.46 mm were used. The tests were conducted in cyclic tension with a cycle frequency of 30 Hz and a stress ratio of R = 0.1. The tests were carried out at a room temperature using an Instron Electropuls E3000 servo-hydraulic machine (Instron, High Wycombe, UK) equipped with Wolpert Wilson Wave Matrix software (Version 2). The experimental error was below 0.25% of the measured value. The fatigue limit, or fatigue strength, σ_R_ was determined. The tests were carried out in accordance with GOST 25.502-79 (Russian Government standard for fatigue strength measurements), which is similar to the ASTM E466 standard (USA). The fatigue limit was defined as the stress amplitude at which at least three samples did not fail after 10^7^ loading cycles.

Biodegradation in vitro was studied by two methods. First, the degradation rate in a saline solution (0.9% NaCl) at 37 °C was determined by the mass loss method. This method is frequently used for assessing the corrosion rate of magnesium alloys due to its simplicity, low cost, and ease of monitoring and parameter control [36]. For that, penny-shaped samples of the alloy in the initial and the final deformed state with a diameter of 10 mm and a thickness of 1.5 mm were ground on abrasive paper (P2500), and then immersed in 200 mL of the solution for one day. Before weighing the specimens after immersion, the degradation products were removed according to ASTM_G1-03-E. Second, the relative mass loss in fetal bovine serum (FBS; Hy Clon, Thermo Fisher, Loughborough, UK) was measured. For this purpose, plates with dimensions of 10 × 5 × 0.3 mm (length × width × thickness) were cut from the alloy in both structural conditions. Before the experiment, the samples were sterilized using 70° ethanol for 18 h, and then dried under sterile conditions. The sterile samples were immersed in 5 mL of FBS poured into sterile containers. The incubation medium was changed every three days. The samples were removed from the medium and analyzed after 2, 4, and 6 weeks of incubation and cleaned from biodegradation products.

The change in mass was determined by weighing the samples on an electronic scale GR 200 (A&D, Tokyo, Japan) with an accuracy of four marks per gram. The relative mass loss (ML) was determined according to the formula:

$$\mathrm{ML}\;=\;\frac{m_{0}-m_{i}}{m_{0}}\times 100\%,$$

where m_0_ and m_i_ are the initial and final mass of the plate, respectively.

**Figure 2:**
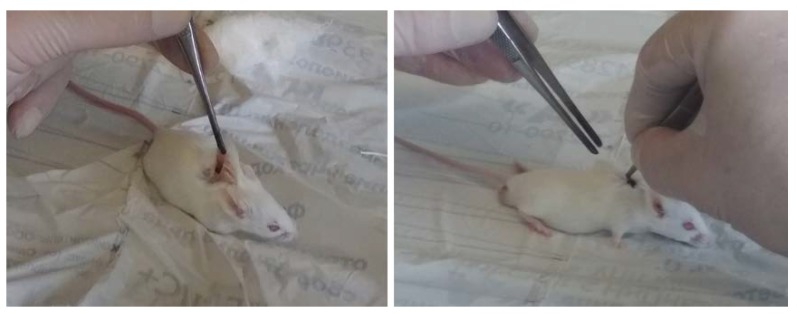
In vivo experiment design.

For in vivo assays, 12 male mice of Balb/c line (with a weight of 20 ± 2 g), divided into two groups (six animals per group), were used as biological objects. The alloy plates used for implantation had the dimensions of 10 × 5 × 0.3 mm. As in the in vitro studies, prior to the tests the samples were sterilized with 70° ethanol for 18 h and then dried under sterile conditions. They were implanted subcutaneously into the back of the mice (one sample per one animal). The edges of the wound were sutured using surgical silk (Figure 2). The mice were maintained under standard conventional conditions (housed in polycarbonate cages, food and water ad libitum, an air temperature of 20 ± 2 °C) before the experiment and during the entire observation period.

During the postoperative period, the biodegradation rate of the sample was monitored by measuring the volume of gas accumulated in the implantation area once every three days. The samples were removed from the animals after 2, 4, and 6 weeks of implantation. After euthanasia, mice underwent autopsy for extraction of the implants and morphological examination of the implantation area to be conducted. The presence of macroscopic signs of rejection, inflammation, injection of blood vessels of the dermis, and subcutaneous fascia was assessed. The samples were removed from the connective tissue capsule after the examination and an assessment of the growth of blood vessels on their surface. Parts of the outer capsule adjacent to the sample were fixed with formalin and stained with hematoxylin–eosin (HE; PanEco, Moscow, Russia) and examined with optical microscope Axiovert 2 (Carl Zeiss, Oberkochen, Germany). The samples were cleaned from biodegradation products and weighed.

The animal test protocols used in this work were evaluated and approved by the Ethics Committee of the N.N. Blokhin National Medical Research Center of Oncology of the Ministry of Health of the Russian Federation (Moscow, Russia; order dated: 2019_5_12; project identification code: АААА-А19-119061190077-2). They are in compliance with the order of the Government of Russian Federation (No. 1172), intergovernmental standard “Principles of good laboratory practice; GLP” (GOST 33647-2015), and the order of the Ministry of Health and Social Development of Russian Federation (No. 708n).

## 3. Results and Discussion

**Figure 3:**
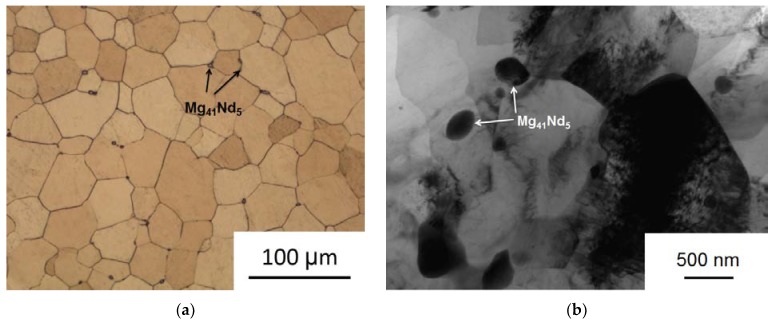
The microstructure of alloy WE43 in the initial (annealed; **a**) and deformed (**b**) states.

The main idea of multiaxial deformation was to use the full potential of dynamic recrystallization for refining the microstructure of the material. Specifically, in this work the intention was to carry out MAD at temperatures and strain rates enabling superplastic deformation of the alloy thus ensuring a high proportion of high-angle grain boundaries [37]. The expectation was that a homogeneous recrystallized structure obtained would radically change the properties of the material, including its in-service performance. This strategy proved to be successful, the multi-pass deformation at elevated temperatures with a step-wise temperature decrements did lead to the formation of a homogeneous ultrafine-grained structure in alloy WE43 (Figure 3).

The initial alloy structure consisted of equiaxed grains of a supersaturated solid solution with an average grain size of 64.9 ± 3.3 µm. Small inclusions of the Mg_41_Nd_5_ phase [38,39] about 2–3 µm in size, which did not completely dissolve during the homogenization process, were also observed. As a result of MAD processing, a rather uniform UFG structure with clearly defined grain boundaries was formed. The average grain size dropped to 0.93–0.29 µm. It should also be noted that the deformation process induced the precipitation from the supersaturated matrix of finely dispersed particles of the Mg_41_Nd_5_ phase with an average size of 0.34 ± 0.21 µm. Precipitation of particles may be associated with the heating prior to MAD and the elevated temperatures during the process. The small size of these particles may be related to the increased density of MAD-induced crystal lattice defects promoting a high density of precipitate nucleation sites. We already observed similar effects in our previous work on high pressure torsion [9] and equal-channel angular pressing [15] of WE43.

The formation of an ultrafine-grained structure and the decomposition of a supersaturated solid solution with the precipitation of particles of the Mg_41_Nd_5_ phase lead to an improvement in the mechanical strength of the alloy. The yield stress (YS) increased significantly—from 161 MPa in the initial state of the alloy to 210 MPa after deformation. The ultimate tensile strength (UTS) also increased from 234 to 300 MPa (Table 1). It should be noted that the increase in the strength characteristics of the alloy owes mainly to the strong microstructure refinement, the contribution to strength from the dispersed precipitate particles being very small. In general, their volume fraction was so small that it could not be determined using quantitative X-ray diffraction analysis.

At the same time, the deformation processing positively affected the ductility of the alloy as represented by the elongation to failure (Table 1). It nearly doubled after MAD deformation. This behavior can be associated with a favorable transformation of the texture caused by the MAD processing. It is common knowledge that texture caused by deformation has a serious effect on the final properties of the magnesium alloys, especially on their ductility. The formation of a favorable texture can result in enhanced ductility despite a strong microstructure refinement. It was shown in early works on magnesium alloys that prismatic slip is often activated during the MAD process while a high propensity for basal slip is retained. In this case, both the prismatic and the inclined basal texture can be formed [23,40]. Hence, it can be conjectured that the high activity of both basal and prismatic slip, as well as the formation of a favorable texture, can be a probable reason for the improvement of tensile ductility of alloy WE43 by MAD.

**Figure 4:**
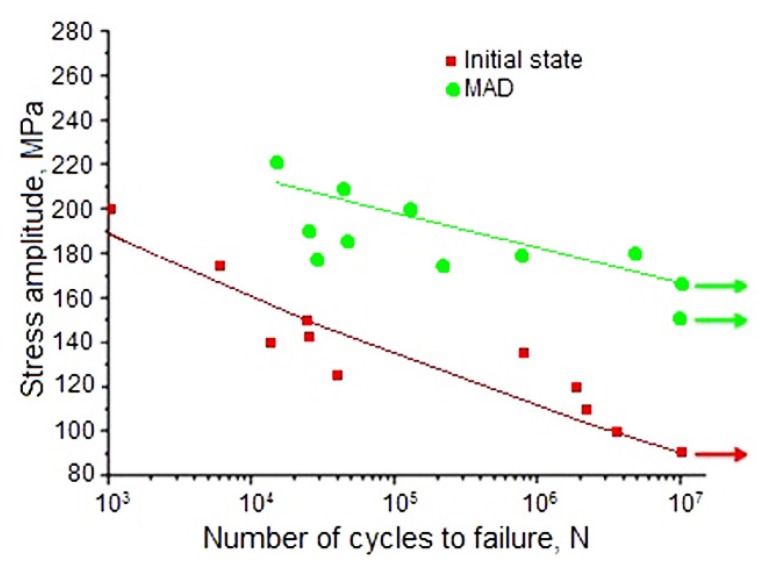
Fatigue behavior of alloy WE43 in the initial and the ultrafine-grained (UFG; MAD) states.

As mentioned in the Introduction, alloy WE43 is a promising candidate material for medical applications, notably in orthopedic implants. Such implants are exposed not only to static, but also to cyclic loads. To characterize the fatigue performance of the alloy in the initial and the UFG states, its fatigue limit was measured (for the number of cycles N = 10^7^). It was shown that MAD-induced grain refinement, along with a significant increase in static strength, also led to an increase in the fatigue limit (σ_R_), which rose from 90 to 165 MPa (Figure 4 and Table 1). It can be concluded that the proposed MAD treatment could significantly increase the ability of the material to resist cyclic loads. This property is especially important in orthopedic surgery, e.g. in screws for fixation of leg and rib fractures. In these cases implants experience cyclic loading during walking or breathing and sufficient fatigue strength is crucial for the structural integrity of the implant. To rationalize the MAD-induced enhancement of the fatigue strength of WE43, a well-established correlation between this quantity and the ultimate tensile strength [41] can be invoked. Apparently, the increase of the latter (cf. Table 1), which is believed to be associated with grain refinement and, to a lesser extent, precipitation of Mg_41_Nd_5_ particles brought about the concomitant increment of the fatigue strength.

The corrosion resistance in the solutions simulating body fluids is another major in-service property of magnesium alloys as medical materials. Magnesium is characterized by an increased degradation rate in a physiological environment, accompanied by intense hydrogen evolution. In this case, hydrogen often accumulates in the areas of implantation, forming cavities filled with gas. These cavities are an additional obstacle to faster healing of the injury, and can also cause complications in the postoperative period. Therefore, a decrease in the degradation rate, which will also lead to a decrease in gas evolution rate, is an important pursuit of research into magnesium alloys as medical materials.

The study of the degradation rate of alloy WE43 in a physiological saline solution 0.9% NaCl showed that MAD deformation inhibited the degradation of the alloy. While the degradation rate was 2.16 ± 0.11 mm/year in the initial state of the alloy, it dropped to 1.58 ± 0.12 mm/year after MAD processing. In the cited article [34], a comparative analysis of the biodegradation rate of homogenized alloy WE43 in bodily fluids in various conditions was conducted. It was established that in vitro biodegradation in FBS was much faster than in vivo, after subcutaneous implantation in test animals. The mentioned study posed questions about the effect of microstructural modification of alloy WE43 on the biodegradation rate and the kinetics of the concomitant gas formation under in vivo conditions. A comparison of our results with literature data confirms that MAD is a promising technique for improving both the strength and the corrosion resistance of alloy WE43. Jiang et al. [42] showed that the degradation rate of extruded WE43, measured in SBF over two days, was ~3.9 mm/year. A similar magnitude of the degradation rate was reported for cast WE43 alloy coated with nano-hydroxyapatite (nHA) particles [43].

**Figure 5:**
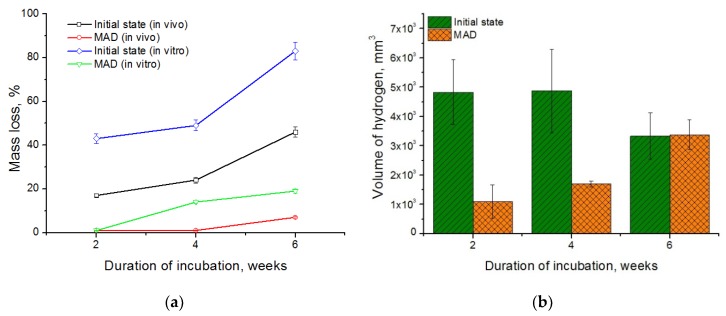
A comparison of the in vitro and in vivo biodegradation rate (**a**), as well as the rate of hydrogen evolution during the incubation in vivo (**b**) of WE43 in the initial and the deformed states.

A comparison of the mass loss of the alloy in the initial and the UFG states measured under in vitro and in vivo conditions is presented in Figure 5a. In vivo studies showed that the biodegradation of the alloy in the UFG state proceeded much slower than in the initial state. Plates cut from the initial alloy fully deteriorated already two weeks after the beginning of the experiment. This rapid degradation of the alloy in the initial state was accompanied with the release of a significant amount of hydrogen gas, as reflected in its accumulation in the tissues surrounding the samples. The process of biodegradation of the alloy in the ultrafine grained state was much slower. A significant accumulation of hydrogen in tissues and erosion of the samples, especially at the edges, was observed over four weeks after implantation. The effect progressed with the implantation time. However, by the end of the observation period the changes in the morphology of the alloy samples after MAD were quite insignificant. Their shape was completely preserved, structural integrity was not lost, and only the color of the surface of the plates changed. The mass loss of the coarse grained alloy amounted to 46% of the initial mass after six weeks of implantation, while the mass loss of the UFG alloy was just 7%. At the same time, the data obtained demonstrates a significant acceleration of the degradation process of the alloy samples under in vitro compared with in vivo conditions, both for the initial and the deformed alloy. Under in vitro conditions, the coarse grained alloy lost almost half of its initial mass already after two weeks of incubation—as opposed to six weeks under in vivo conditions. The mass loss of the initial alloy was about 83% by the end of the experiment. These data are consistent with the results of the cited article [34] where a comparative analysis of the biodegradation rate of homogenized alloy WE43 in bodily fluids in various conditions was presented. It was established that in vitro biodegradation in FBS was much faster, and was accompanied with a much vigorous gas formation, than in vivo.

The degradation process of the alloy after MAD was markedly slower than in the initial state at all times. In particular, the degradation rate of the MAD-treated alloy under in vitro conditions was ~1%, 14%, and 19% after 2, 4, and 6 weeks of incubation, respectively, and only 7% after 6 weeks of implantation in vivo. The cause of the difference in the biodegradation rate of the alloys under different conditions can be a different protein and gas composition of the incubation medium due to the large contact areawith atmospheric air during incubation in vitro.

Moreover, it is well known that in aqueous solutions, magnesium and its alloys corrode with the evolution of hydrogen [44]:

Mg + 2H_2_O → Mg^2+^ + 2OH^−^ + H_2_↑. (1)

Therefore, a decrease in the degradation rate of the alloy should be accompanied with a decrease in the rate of release of hydrogen into the tissues surrounding the implant. Our results do confirm this. Figure 5b shows that a reduction of the degradation rate achieved through grain refinement by MAD found in vivo slows down the rate of gas formation. For comparison: the volume of the gas bubble formed in the tissues around the plate cut from the initial alloy after the first two weeks of implantation was 4828 ± 1107 mm^3^, while that of the bubble around the MAD-treated alloy plate was 1101 ± 567 mm^3^. After four weeks of implantation, the volume of the gas bubbles did not change noticeably and amounted to 4878 ± 1422 mm^3^ and 1700 ± 100 mm^3^ for the initial and deformed (UFG) states, respectively. Extension of the implantation time to six weeks led to growth of the volume of the gas bubble for the alloy in the UFG state (to 3381 ± 508 mm^3^), obviously due to the onset of active degradation. Moreover, this quantity did not change for the initial state of the alloy and amounted to 3329 ± 788 mm^3^. It should be noted that the volume of gas bubbles was nearly the same for both states of the alloy after six weeks of implantation. However, it should be borne in mind that the samples cut from the coarse grained alloy in the initial state decayed almost completely after six weeks of implantation (the mass loss was ~46% of the initial mass, as mentioned above), whereas the samples made from the deformed alloy exhibited almost no changes (the relative mass loss being as low as ~7%).

Based on the data obtained, it can be concluded that MAD makes it possible to significantly reduce the biodegradation rate of magnesium alloy WE43 both under in vitro and in vivo conditions. This behavior can be caused by the formation after MAD of a fairly homogeneous UFG structure with a small amount of finely dispersed corrosion-resistant Mg_41_Nd_5_ phase, which did not give rise to any significant corrosion pair formation. Moreover, the generation of numerous crystal lattice defects (dislocations, grain boundaries, etc.), which constitute short-cut diffusion paths, can be the reason for an increase of the degradation rate in early stages of the tests that led to rapid formation of a passivation film of corrosion products on the specimen surface. Once formed, this film may have provided an effective barrier retarding further biocorrosion [45]. Studies of the composition of corrosion products were carried out previously in many works. Ascencio et al. [46] discovered that magnesium hydroxide Mg(OH)_2_ was mainly formed on the surface of the samples during degradation process in a modified simulated body fluid solution (m-SBF) [47] comprising a mixture of different salts. In the case of incubation in Dulbecco’s modified eagle medium (DMEM), two layers are formed sequentially. The first layer is adjacent to the metal and consists of a mixture of Mg(OH)_2_ and (Mg,Ca)CO_3_, and the second one is enriched in Ca and P(a Ca-phosphate layer) [48]. Liu et al. [49] investigated the corrosion behavior of pure Mg and alloy WE43 in human bile for 60 days. They showed that a Mg(H_2_PO_4_)_2_ layer formed on the specimen surface during the corrosion process, which is a consequence of the complex composition of the corrosive medium. Quite generally, the composition of the biodegradation products depends directly on that of the medium. However, in all cases studied, the biodegradation process was accompanied with the formation of a protective layer on the specimen surface, which retarded further degradation of the alloy.

**Figure 6:**
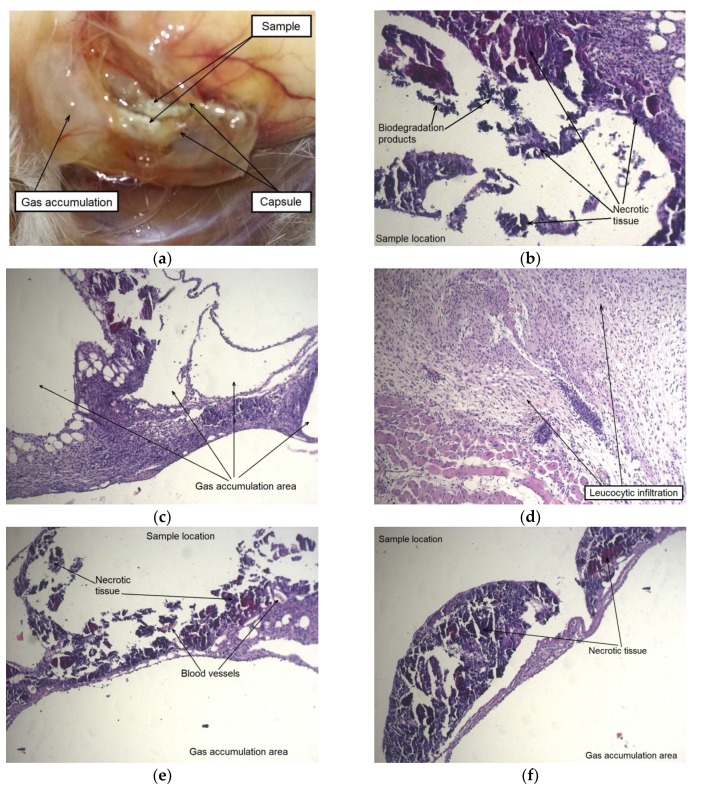
The sample of alloy WE43 in the initial state and the area around the implant two weeks after surgery: fragments of the sample inside the capsule, swelling and accumulation of gases in the surrounding tissue (**a**), results of histological examination of mouse tissues (hematoxylin–eosin (HE) staining) in the area of contact with the samples, showing the formation of large gas cavities, the expansion of blood vessels, the accumulation of biodegradation products, leucocyte infiltration, and contact tissue necrosis (**b**–**f**).

To assess the effect of the alloy in the initial and deformed states on animals, a histological examination of the surrounding tissues was performed. A morphological study showed that after two weeks following implantation a thick tissue capsule was formed around the sample in the area of the surgical operation in the case when the implanted alloy WE43 was in the initial state. An accumulation of gas was detected in one or more cavities inside the capsule and in the tissues surrounding the alloy sample. The capsule around the sample was permeated with dilated vessels and capillaries, and the tissues around the implantation area were swollen (Figure 6).

**Figure 7:**
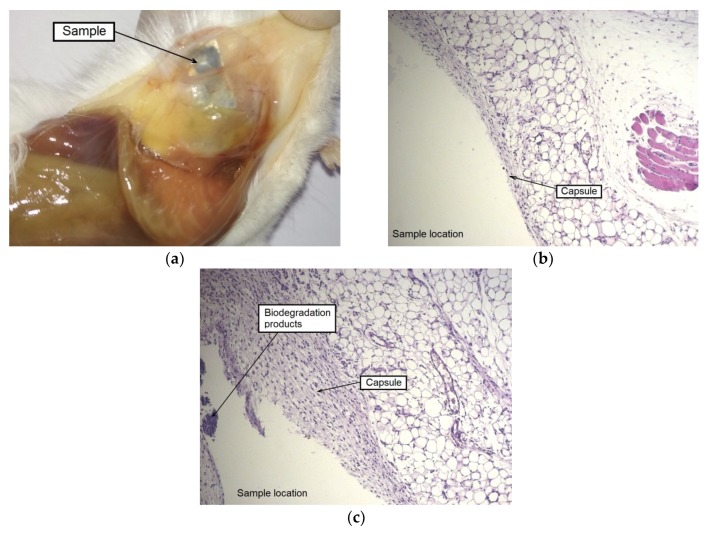
A sample of the alloy WE43 in UFG state and the area around the implantation after two weeks after surgery: the alloy sample with minor signs of degradation, retaining its integrity in the surrounding tissue without signs of gas accumulation and inflammation (**a**), result of a histological examination of tissues of a mouse (HE staining) in the area of contact with a sample demonstrating the formation of a thin-walled capsule around the sample with minor signs of accumulation of biodegradation products and the absence of signs of gas accumulation (**b**,**c**).

The autopsy results for a group of animals with an implanted alloy WE43 in the UFG state also showed the formation of a capsule around an implant. However, there were no signs of local edema or increased blood flow in this area. No presence of dilated blood vessels, hemorrhages, and gas accumulation was also detected. A histological examination of tissues in the area of contact with the MAD-treated UFG alloy sample showed that a large part of the sample was surrounded by a thin-walled capsule without signs of general cellular infiltration. It can be concluded that the process of biodegradation of the sample for the period (two weeks) occurred mainly at its corners. This is supported by the observed localized thickening of the capsule with the attraction of leucocytes near the corners of the sample, as well as by the presence of degradation products from the inside of the capsule (Figure 7).

**Figure 8:**
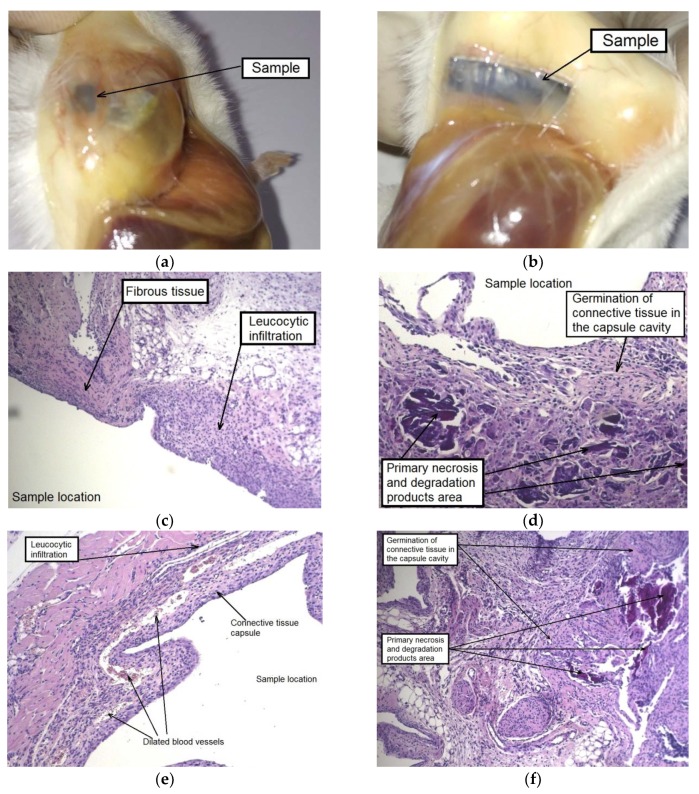
Samples of alloy WE43 and the implantation areas four weeks after surgery: alloy samples in the initial (**a**) and the UFG (**b**) states with the surrounding tissue; result of a histological examination of tissues (HE staining) in the contact area with the alloy in the initial (**c**,**d**) and the UFG (**e,f**) states.

The autopsy of animals of both groups, carried out after four weeks of implantation, showed a noticeable disintegration of the alloy samples in the initial state and a much less pronounced degradation of the MAD-processed alloy. The formation of a thick-walled fibrous capsule, abundantly permeated with blood vessels, was detected around the alloy sample in the initial state. Hyperplasia of the subcutaneous fascia adjacent to the sample was also observed in the implantation area. Moreover, signs of severe alloy degradation with loss of the integrity of the plate were detected on the entire surface of the samples tested and, in particular, on their periphery (Figure 8a).

As for the alloy in the UFG state, the accumulation of a small amount of gas inside the capsule formed around the sample was observed. However, there were no indications of tissue edema and the occurrence of granulation in the implantation area. The samples retained their integrity, but completely changed their color to dark, which indicates the beginning of degradation processes and the formation of an oxide film (Figure 8b).

A histological examination showed a similar picture for the contact area of the animals’ tissues with both types of samples: a thick-walled capsule from connective tissue fibers permeated with blood vessels adhered to the sample, and cell infiltration was observed around it involving giant multinucleated cells. The foci of necrosis resulting from contact with both types of alloy samples at the initial stage of implantation and alloy degradation products were immersed in the newly formed connective tissue, which sprouted into the capsule cavity and began to gradually replace the volume of the subcutaneous cavity that was created for the implantation of the alloy sample (Figure 8c–f). A similar picture was observed in our earlier work [34] in which a histological analysis of tissues surrounding a homogenized WE43 implant was conducted after two months of subcutaneous implantation. As in the current assay, a fibrous capsule permeated with profuse visible blood vessels was formed. It is interesting to note that, as distinct from the in vitro behavior in FBS, the specimens implanted in mice degraded at a much lower rate.

**Figure 9:**
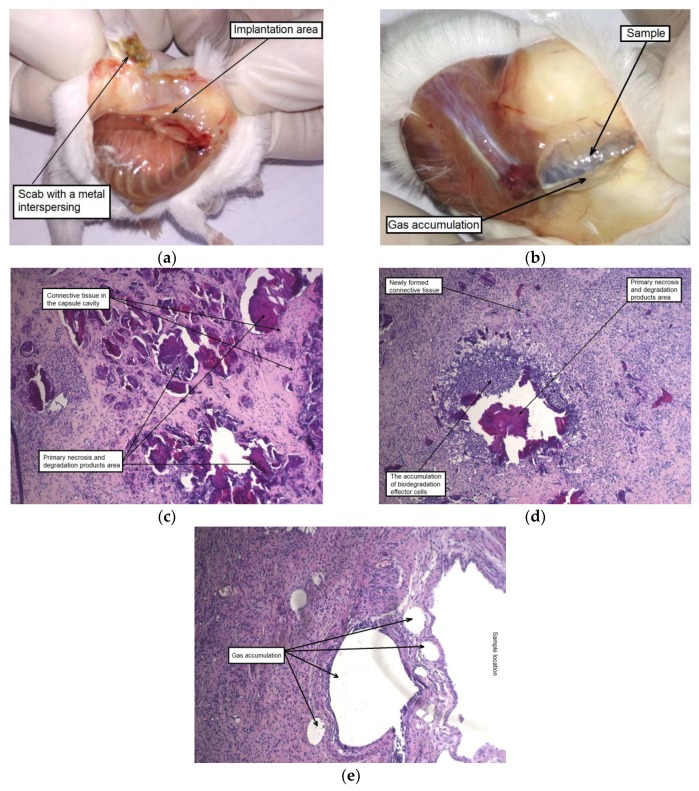
Samples of alloy WE43 and tissues of the implantation area six weeks after surgery: complete degradation of the sample in the initial state of the alloy, a small amount of biodegradation products of the alloy was found only in the skin scab (**a**); capsule formation around the sample in the UFG state of the alloy and gas accumulation in its cavity (**b**); the results of histological examination of mouse tissues (HE staining) in the area of contact with the samples in the initial (**c**,**d**) and the MAD-processed (**e**) states of the alloy.

After six weeks of implantation, the original plate shape was no longer detectable in the case of the alloy WE43 in the initial state. Their fragments in form of dispersed small particles were found in the cutaneous scab. The subcutaneous fascia in the area of implantation was significantly hyperemic and foci of connective tissue hyperplasia were observed. By contrast, the alloy samples in the UFG state did retain their structural integrity. Signs of insignificant degradation were observed only along the edges of the UFG alloy plates. The MAD-treated alloy samples were surrounded by a fibrous capsule in which gas accumulation was detected. It should also be noted a dilation of individual blood vessels within the area of implantation was observed (Figure 9a,b).

A histological examination of animal tissues after implantation of MAD-treated alloy samples revealed an accumulation of a significant amount of gas not only in the capsule cavity surrounding the sample, but also in its walls and the adjacent tissues, which were profusely permeated with histiocytes (Figure 9e). A significant amount of small isolated foci of the biodegradation products and necrotic tissue, surrounded by a cell bank and included in the newly formed fibrous connective tissue filling the volume of the cavity around the implant was also observed after six weeks. Thus, it could be concluded that recovery processes occurred once signs of active degradation and accompanying gas formation in animal tissues disappeared (Figure 9c,d).

A main outcome of the experiments in vivo was the finding of a significant reduction in the biodegradation rate for the alloy WE43 in the UFG state compared to its biodegradation behavior in the initial state. However, according to the results of histological studies the same sequence of local reactions of the organism of mice to the implants in the two different states considered was found. What is important for potential applications of the alloy as a base of an implanted medical device is that these reactions proceeded at different speeds depending on its structural state. In particular, the intense cell infiltration and blood vessel permeation of tissue around the alloy in the initial state in combination with multiple foci of necrosis was observed already as early as two weeks after surgery. For comparison, a similar morphological picture around the MAD-treated alloy was observed after four weeks. The replacement of the newly formed fibrous connective scar tissue of the artificially formed cavity in which the implants were placed and defects of adjacent tissues caused by gas formation and necrosis was detected after six weeks of monitoring both groups of animals.

In summary, a local toxic effect in the form of hydrogen gas accumulation in the interstitial space and the formation of necrotic masses in the contact area were observed as a result of implantation of alloy WE43 in both states. However, the foci of gas accumulation disappeared, and necrotic masses were partially replaced with newly formed connective tissue after quite a short time. In other words, intensive reparative processes leading to the replacement of the implant and the cavity around it were found to occur in the area of implantation.

## 4. Conclusions

1. The multiaxial deformation of the alloy WE43 led to a significant refinement of the structure down to an ultrafine-grained state with an average grain size of 0.93 ± 0.29 μm. Fine particles of the Mg_41_Nd_5_ phase with an average size of 0.34 ± 0.21 μm were also formed as a result of MAD.

2. The UFG structure significantly strengthened the alloy, increasing its ultimate tensile strength from 234 MPa in the initial state to 300 MPa after deformation. In this case, a significant increase in the total elongation of the alloy from 9% to 17.2% was also observed, apparently due to the favorable texture formed by MAD processing.

3. An additional positive effect of the formation of the UFG structure was an increase in the fatigue limit of the alloy WE43 from 90 to 165 MPa.

4. UFG structure induced by MAD processing led to an increase in the corrosion resistance of WE43. The degradation rate of the UFG alloy in a 0.9% NaCl solution decreased from 2.16 ± 0.11 mm/year to 1.58 ± 0.12 mm/year. This was consistent with a significant decrease in the relative mass loss observed under both in vitro and in vivo conditions. The drop of the relative mass loss after six weeks of experiment in vitro (83% and 19% for the initial and the UFG alloy, respectively) and in vivo (46% and 7%, respectively) was impressive. In addition, inhibition of gas formation associated with a decrease in the in vivo biodegradation rate for UFG alloy was a further beneficial effect of MAD processing.

5. Some negative side effects of implantation, such as gas accumulation, as well as accumulation of biodegradation products and necrotic tissue masses in the contact area were observed. These processes were almost twice as fast for the alloy in the initial state compared with the alloy with UFG structure, which speaks in favor of MAD processing. On a positive side, the foci of gas accumulation disappeared, and necrotic masses were replaced with newly formed connective tissue towards the end of the biodegradation process. As a result, the development of intensive reparative processes leading to the replacement of the implant and the cavity around it with the connective tissue was observed.

6. The entity of the data obtained suggest that processing of alloy WE43 by multiaxial deformation was a promising avenue for improving the performance of the alloy in future medical implant applications.

## Figures and Tables

**Table 1:** Mechanical properties of alloy WE43 in the initial and post-multiaxial deformation (MAD) states.

Processing	YS, MPa	UTS, MPa	El, %	σ_R_, MPa
Initial state	161	234	9	90
MAD	210	300	17.2	165
